# Hybrid deep learning model for multimodal vocal and lung signal analysis in health monitoring

**DOI:** 10.1038/s41598-025-32779-0

**Published:** 2026-05-02

**Authors:** S. Revathi, K. Mohanasundaram, Palanichamy Naveen

**Affiliations:** 1 Department of Electrical and Electronics Engineering, KPR Institute of Engineering and Technology, Coimbatore, India; 2 Department of Electronics and Communication Engineering, Dr. N.G.P. Institute of Technology, Coimbatore, India

**Keywords:** Lung signal, Vocal signal, CBiRNN, Ensemble method, Healthcare, Biomedical engineering, Machine learning

## Abstract

Non-invasive health monitoring has recently gained a lot of consideration in the modern healthcare system, because it has the potential to diagnose diseases earlier and can monitor patients in a remote manner. This research presents a hybrid approach in healthcare monitoring by integrating vocal and lung abnormality detection using a multinetwork model. The model utilizes multiple data sources and Mel Frequency Cepstral Coefficients (MFCCs) to capture the frequency spectrum of the signal. A multinetwork model developed for disease identification is made up of hybrid deep learning networks, which consist of Convolutional Neural Networks (CNN) and Bi-directional Recurrent Neural Networks (BiRNN) referred as the Convolutional Bi-directional Recurrent Neural Network (CBiRNN). These CBiRNN models process both the vocal and lung datasets in parallel and feed the predicted results into the ensemble model for comprehensive evaluation. The experimental results show that the proposed CBiRNN model achieves 92% accuracy in voice disorder detection and 98% accuracy in respiratory disorder detection, while the ensemble model attains 98% accuracy for both voice and lung prediction. This innovative multimodal processing technique demonstrates significant potential in advancing health monitoring systems, offering a pathway to more accurate and reliable diagnostic tools.

## Introduction

Automatic pathological identification through non-invasive approaches represents one of the most innovative developments in clinical environments for early disease detection and medical conditions in a remote monitoring system. With the help of Artificial Intelligence (AI) in the medical field, these innovative practices can achieve high accuracy in identifying pathological conditions. In December 2023, Los Angeles Pacific University published a career blog about AI applications in the healthcare industry^1^, highlighting how AI is being used for automatic speech recognition, disease identification and efficient medical information for precision medication and early diagnosis. Speech analysis is emerged as a predominant tool for identifying pathological conditions because it reflects both physical and psychological states. A person’s speech patterns and voice characteristics reveal various medical conditions, where there is any alteration in voice signal such as loudness, pitch, clarity and fluency. This alteration makes speech analysis a valuable diagnostic approach in identifying several medical conditions, especially in vocal and lung diseases.

For diagnostic diseases, speech signals for various pathological conditions are publicly available in a database format, where it contains pathological and normal speech signals. Hussein et al.^2^ developed the diseased signal classification using the Saarbruecken Voice database (SVD) and it shows the best accuracy for the SVM algorithm. Some glottal features are also used for the classification of normal and pathological voice by means of using the Massachusetts Eye and Ear Infirmary (MEEI) database^3^. Voice Icar fEDerico (VOICED) dataset is used in the assessment of voice disorder by recording the voice sample^4^. These vocal signals can automatically detect vocal and lung diseases by training the deep learning models using acoustic features. There are several benefits of using speech signal analysis in clinical practice, such as early detection, non-invasive diagnostics, remote monitoring, and continuous tracking. The contribution of research work is highlighted as follows:

- Research work is performed using Multiple data sources such as vocal and lung datasets, which were augmented using signal augmentation techniques.
- Acoustic characteristics are extracted from the vocal and lung dataset using Mel Frequency Cepstral Coefficients (MFCCs) to obtain the frequency spectrum from the speech signal.
- A multinetwork model is used to classify the pathological signal from the normal signal, which combines the analysis of vocal and lung signals using a hybrid Convolutional Neural Network and Bi-directional Recurrent Neural Network (BiRNN) model.
- The predictions from the vocal and lung models are fed into an ensemble model for a comprehensive evaluation.

By leveraging complementary data from both sources, this combined analysis provides a more thorough assessment and may help in identifying abnormalities. This innovative multimodal signal processing technique paves the way for more advanced health monitoring systems. The research paper is organized as Sect. 1 focuses on research motivation and key contributions, Sect. 2 discusses the background study and the related work on vocal and lung disease identification using speech signals, Sect. 3 presents the dataset used, feature extraction and the proposed CBiRNN network in classifying the various diseases, Sect. 4 shows the model performances in classifying the voice and lung signal using a multimodal network and finally, Sect. 5 concludes the work performed.

## Background

In neurological speech disorder classification, wavelet scattering transform feature extraction techniques were used to get higher efficiency in his work, combined with Mel Frequency Cepstral Coefficients (MFCC) for the machine learning algorithm^5^. Mujeeb Ur Rehman et al.^6^ conducted the study with various Machine learning models using the jitter, shimmer, fundamental frequency, and periodicity in the dataset to segregate the signal in the form of healthy and pathological signals by extracting the necessary aspects of quality and its associated characteristic condition. To detect the glottal pathology, the extraction zero-crossing rate along with Mel-frequency filter bank cepstral coefficients can give a solution of disorder at low cost^7^. By cascading these CNN-RNN models, high accuracy can be obtained and by incorporating the CNN-Bidirectional long short-term memory (BiLSTM) network, voice disorder can be classified from speech recording^8^. Farazi. S and Shekofteh. Y^9^ presented the pathological detection Convolutional Neural Networks (CNN) model for spontaneous speech data by training on MFCC and Mel spectrogram features.

Art of voice disorder detection states the critical difference between the healthy and pathological speech using the self-supervised representation, such as Wav2vec2.0 and HuBERT^10^ and reduces the gradient loss and increases the prediction. Even ResNet34 a pre-trained CNN model can classify the signal in the form healthy and abnormal signal^11^. Multiple machine learning algorithms, such as random forest and support vector machine are used to identify speech problems^6^. P.V.L. Narasimha Rao and S. Meher^12^ implement a three-stage classification system employing ResNet, GoogleNet and Radial basis function-gated Recurrent Unit. Octave changes, such as a monotone voice, might indicate neurological diseases or emotional flatness, whilst irregular pitch ranges may indicate hormonal imbalances or vocal cord problems. Laryngeal disorders can be classified using a Support Vector Machine (SVM), Gradient-Boosting Machine (LightGBM), Artificial Neural Network (ANN) and CNN to identify vocal cord paralysis and innocuous mucosal diseases using speech data^13,14^ and to classify the age & gender using a speech data neural network developed by Dhanalakshmi et al.,^15^.

Other medical conditions, such as chronic obstructive pulmonary disease (COPD), asthma, or COVID-19, can cause voice hoarseness and interruption. Lung model performance is evaluated using the deep learning network under variable parameters for lung sounds^16^. Ahmad H. Sabry et al.,^17^ presented a discussion on Cognitive respiratory diseases easily using an audio signal using a machine learning algorithm. With the help of infant crying data, critical pediatric health conditions were indirectly identified using an audio spectrogram and transformer-based algorithms^18^. Lung illnesses can be identified using machine learning to analyze acoustic signals^17^ and Jiakun Shen et al.,^19^ used Resnet18 to classify respiratory diseases based on cough sound data. Patients with pulmonary tuberculosis can be identified using the feature method based on cough sound signal^20^ and the emotion of a crying infant can be detected using the whale optimization algorithm^21^. A novel convolutional hidden Markov neural network^22^ is suggested for automated pulmonary disease identification and infected region recognition. Even breath can be analyzed for early diagnosis of lung cancer by using a convolutional neural network (CNN) designed by Byeongju Lee et al.,^23^. Multi-task learning is used for the simultaneous classification of lung diseases by CNN, MobileNet and Densenet from the International Conference on Biomedical and Health Informatics (ICBHI 2017). Chronic Obstructive Pulmonary Disease and its severity level are classified by using the Resnet 50 model from RespiratoryDatabase@TR^24^.

### Related work

Research on voice and lung pathological detection emphasizes the use of deep learning and machine learning techniques for accurate classification in a non-invasive format.

#### Vocal pathology

A wider number of publicly available databases are employed to train and test the automatic voice disorder detection model are Saarbruecken Voice database (SVD)^25^ is used for CNN-RNN for voice pathological detection, Massachusetts Eye and Ear Infirmary (MEEI) database, and Voice Icar fEDerico (VOICED). For the signal classification feature extraction plays an important role, so the best feature extraction techniques as to be selected. Most commonly, Mel-frequency Cepstral Coefficients (MFCC) are used in audio analysis to extract features in the frequency domain. A study was conducted to examine the Mel-Frequency Cepstral Coefficient robustness on various classifiers^26^ and deep transfer learning is used for voice disorder prediction and classification^27^.

Haydar Ankishana and Sitki Cagdas Inam^28^ introduced the feature level fusion and decision-level fusion methods to detect the voice disorder conventionally by using a convolutional neural network and LSTM for decision prediction. A speech signal is a time-varying signal, it varies depending on the time, with the amplitude of the signal changes due to the pitch of the voice in the patient. Speech data is balanced by using the synthetic minority oversampling technique (SMOTE) and a Convolution neural network is used to detect pathological signal from normal signal^29^. The multiclass classification of voice signals on various stacked vowels is demonstrated in Liu et al.,^30^ and transformer-based algorithms^31^ are used to classify the pathological categories.

#### Lung pathology

Lung pathological conditions such as Chronic Obstructive Pulmonary Disease (COPD), asthma and pneumonia can be identified using sound signals^32^ and the CNN model is used to predict lung diseases^33^. Even cough sounds can also be used for respiratory pathological identification using deep neural networks^34^ and a deep ensemble network is used for abnormality identification as proposed in Wall et al.,^35^. Faezeh Majzoobi et al.,^36^ presented a Convolutional long short-term memory for reliable diagnostics of pulmonary ailments employing several channels for asthma and Chronic Obstructive Pulmonary Disease (COPD), as well as identifying the healthy condition. Life-threatening respiratory problems and respiratory diseases such as COPD can be classified by extracting the features such as MFCC, Chroma, Contract, Mel, and Tonnetz to classify whether the signal has normal or abnormal^37^ and to balance the input data Synthetic Minority Oversampling Technique (SMOTE) algorithm is used. Utilizing Convolutional Neural Network (CNN) lung diseases can be classified by applying the augmentation technic to achieve high accuracy^38^. Borwankar et al.,^39^ proposed an improvised version of a multilayer convolutional neural network for respiratory pathological classification and a parallel convolutional-based autoencoder was developed by Khan et al.,^40^ for pulmonary disease detection.

The existing studies of vocal and lung diseases using various neural network models typically focused on detecting either voice disorders or lung conditions in isolation. So, this work breaks new ground by developing a unified model capable of simultaneously identifying and classifying both types of disease. This innovative approach acknowledges the physiological connection between the vocal and pulmonary systems, potentially enabling more comprehensive and efficient screening processes. By analyzing speech patterns for indicators of both voice and respiratory conditions within a single diagnostic framework, the model offers healthcare providers a more holistic view of patient respiratory health.

## Materials and methods

### Multiple data sources

####  Saarbruecken voice database (SVD)

Saarbreuecken Voice Database is a collection of voice recordings from people with a variety of vocal abnormalities, prepared by phoneticians at the University of Saarland in Germany. It includes vowel a, i, u pronunciation in high, low and high-low formats, along with the German line “Guten Morgen, wie Geht es Ihnen?” at 50 kHz and 16-bit resolution. The database can be publicly accessible^41^ by navigating the search window by selecting the parameter age, gender, voice pathological condition. In this study, five pathological states are used for classification such as 700 dysphonia, 770 functional dysphonia, 2047 laryngeal nerve palsy, 900 laryngitis, and 864 Spasmodic dysphonia along with 4383 healthy samples in .wav format at the bit rate of 800kbps (kilobits per second).

#### Respiratory sound database

Two study teams in Portugal and Greece generated a respiratory sound database, which contains sounds emitted when a person breathes in and out. It contains 920 annotated recordings in duration from 10 to 90 s. These recordings were made over 5.5 h by 126 people and included crackles and wheezes made while breathing^42^. This study uses wheezes and crackles of 480 COPD (Chronic Obstructive Pulmonary disease) and 37 pneumonia patients, as well as 35 normal breathing sounds in .wav format at bit rate of 1058 kbps.

### Augmentation techniques

The dataset used for classifying vocal and lung pathological signals has a different number of samples across the classes; to overcome this imbalance, a data augmentation technique is applied. This technique generates fresh training samples by applying minor deformations to annotated training data while preserving their labels. The method creates new vocal and lung samples through label-preserving transformations of both diseased and healthy speech samples from an existing dataset. The existing collection of vocal and lung samples is referred to as the original data, while newly created data is termed augmentation data^43^. Seven data augmentation methods are utilized to yield an augmented dataset by using augmentation methods such as adding noise, time shifting, altering pitch, stretching audio, random erase, changing speed, and combining two augmentation signals.

#### Noise addition

This is a recognized method utilized to enhance the dataset by artificially injecting different types of noise into the clean speech signal. In this work, random Gaussian noise is added to the original signal by constructing a noise array of the same length and using np.random to generate augmented data scaled by a noise factor of 0.005. By doing so, it boosts the quantity and diversity of the existing training data, hence improving model robustness in data.

#### Time shifting

Time shifting is an augmentation technique in which the audio signal is randomly shifted to the left or right along the time axis to simulate differences in the timing at which the audio is presented. It chooses a 40% shift amount at random (τ) and applies the np.roll function of circular shifting the data. This systematic alternation enables the model to learn representations that are less sensitive to precise timing, which can be vital for distinguishing between healthy and pathological signals.

#### Change of pitch

This method involves altering the fundamental frequency (pitch) of the audio signal to introduce variances that can increase the model’s capacity to generalize. This method utilizes librosa to modify the pitch of an audio signal at random.

#### Time stretching

Time stretching is a technique for modifying the duration or tempo of an audio signal without modifying its pitch. The original data is stretched at a rate of 1.1 intervals in a faster format, causing the signal to change in speaking speed or tempo.

#### Random erase

This augmentation strategy enhances the robustness and generalization of machine learning models. This strategy randomly erases the portions of original data, which can help to prevent overfitting while simultaneously improving performance on unknown data at the rate of 0.1.

#### Speed tuning

Speed tuning is like the stretch function but typically uses more extreme rate values. It changes both the speed and pitch of the audio, simulating faster or slower playback. The original data is tuned at the time stretching rate of 2.

#### Mixup

This technique linearly combines the input with another sample from the original dataset. It combines the signal pair at a rate determined by the parameter to generate a new sample. The mixing ratio of the two signals is from a beta distribution parameter of 0.2 to boost the model’s performance.

### Data pre-processing

The augmented and raw acoustic signal is more challenging in classifying the pathological signal, because it consists of noise and abnormal peaks. To address this issue, signals must undergo pre-processing before being fed to the model. Most commonly, Mel Frequency Cepstral Coefficient (MFCC) is used as an audio pre-processing technique, these coefficients effectively capture the spectral characteristics from vocal and lung signals, making them valuable for tasks such as disease detection^44^. The MFCC extraction process follows a systematic approach: first, a high-pass filter is applied to the input and augmented audio signal to generate a pre-emphasized signal. This pre-emphasized audio signal is then fragmented into small overlapping stationary frames and each frame is multiplied by a Hamming window to smooth the signal and minimize discontinuities at the boundaries. Finally, the windowed frames are transformed into the frequency domain, enabling detailed analysis of the frequency content within the audio signals.

This spectral signal is then filtered using a Mel-scale filter bank, which consists of M triangular filters spaced according to the Mel scale. This stage is crucial because it mimics the human auditory system’s non-linear frequency perception, providing better resolution at lower frequencies. The outputs from these Mel filters are logarithmically compressed to reflect human perception of loudness through filterbank energies. Finally, an Inverse Discrete Fourier Transform is applied to the log filterbank energies, compressing the information into lower-order coefficients. The resulting MFCC features provide a concise, perceptually suitable representation of the audio signal’s spectral properties. The entire procedure, from framing to final coefficient computation, is repeated for each short audio segment, yielding a series of MFCC vectors that capture the evolution of spectral features over time.

### Multinetwork model

**Fig. 1:**
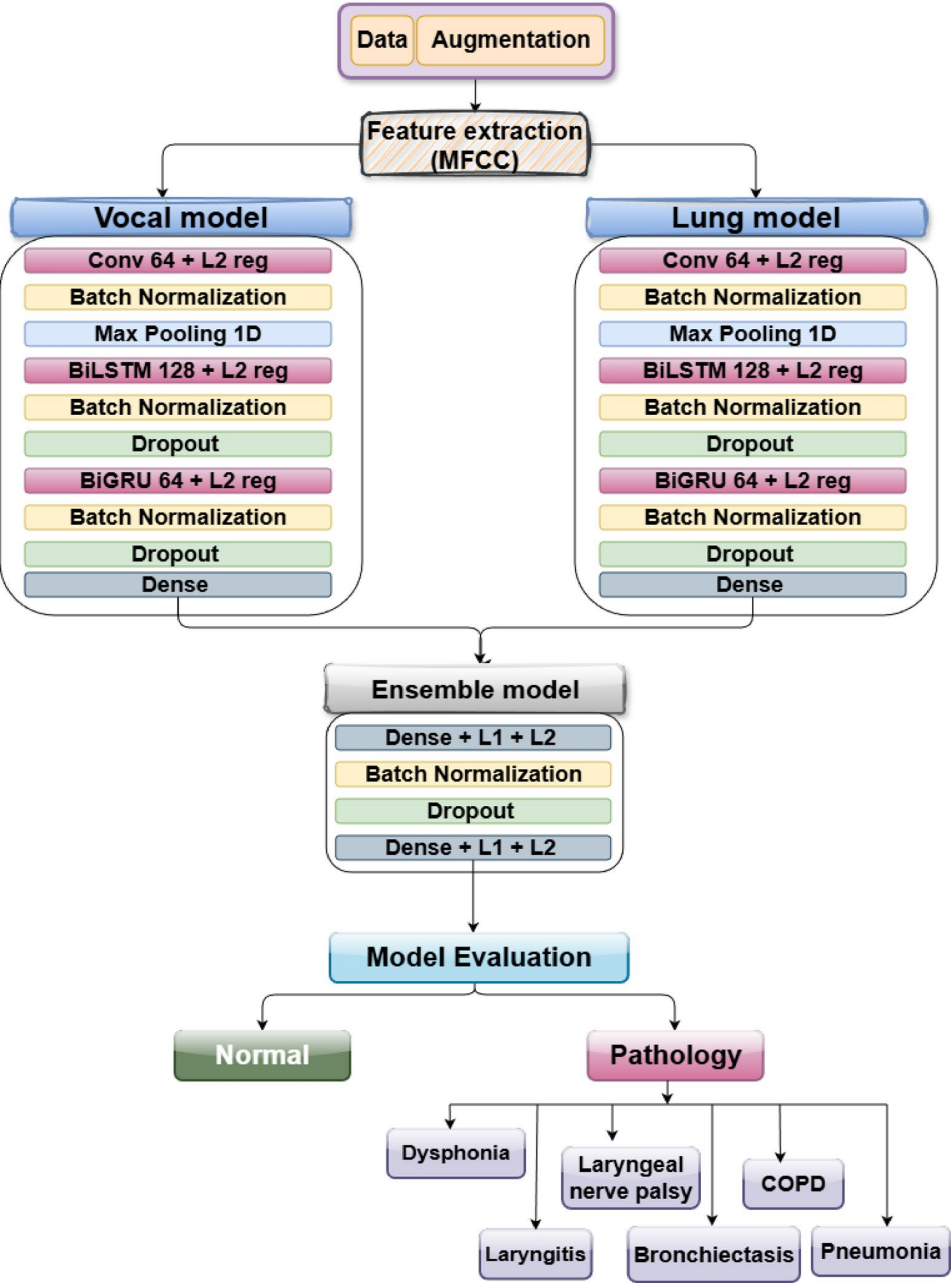
Proposed multinetwork model.

In this innovative approach, hybrid and ensemble models are employed to identify the abnormal signal. For this work, three models that encompass vocal and lung data for comprehensive analysis are proposed and it is explained in Fig. 1. Before being fed into the model, the dataset was separated into three sets: training, testing, and validation, with approximately 20% allocated for testing and 20% for validation from the whole dataset.

#### CBiRNN model

This study proposes two CBiRNN architectures^45^ by modifying each network layer from the work^46^. This model processes the input signal in parallel form and feeds their predictions to an ensemble model for final analysis. The model is made of a hybrid network, which combines a convolutional neural network and a recurrent neural network to improve feature extraction and temporal dependence. The first CBiRNN model processes the vocal data after effective spectral frequency extraction from vocal signals to classify the pathological signal. The model architecture begins with a 1D convolutional layer with 64 filters, which is then batch normalized to ensure training stability. A max pooling layer is used to reduce spatial dimensions and extract the most important characteristics.

The network’s core is made up of two bidirectional layers with 256 and 128 units respectively, which allows the model to capture context from previous and future states in the sequence. These bidirectional RNN layers are followed by batch normalization, dropout, and regularization to improve training stability and prevent overfitting. The parameters used for designing the CBiRNN model are illustrated in Table 1 with their hyperparameter values.

**Table 1:** Various parameters of CBiRNN models.

Layer name	Model hyperparameter	Output shape	Parameter
Input_layer	–	None, 13,1	0
Conv1D + normalization	Unit = 64, active = relu, L2 = 0.01	None,11,64	256
Max_pooling1D	Pool size = 2, Strides = 2	None, 5, 64	0
Bidirectional LSTM + normalization	Unit = 128, Return = True, L2 = 0.01	None, 5, 256	197,632
Dropout	(0.3)	None, 5, 256	0
Bidirectional GRU + normalization	Unit = 64, L2 = 0.01	None, 128	123,648
Dropout	(0.3)	None, 128	0
Dense	1, activation = sigmoid, L2 = 0.01	None, 1	129

The second CBiRNN model is structurally like the first model, it processes lung data using effective feature extraction approaches before feeding it through the CNN-BiRNN pipeline. Both models produce vectors for their respective signal-based pathology markers. The ablation studies were performed to analyze whether our model structure is superior to other works. The studies showed that a model with only convolutional layers achieved 65% classification accuracy. When CNN and LSTM networks were combined, accuracy increased to 85%, though this configuration required the highest training time. Finally, when the model was enhanced with GRU components, results showed reduced training time while maintaining high accuracy.

The optimum hyperparameters of this model, such as batch size, epochs, dropout rate, and learning rate, are determined through fine-tuning with a grid search technique. The optimal parameters are selected based on high test and validation accuracy with minimal difference between them, low test loss, and efficient training time. An Intel Core i7-13700 system with 16 GB of memory was used to train the network. Table 2 presents the optimal hyperparameters for the vocal and lung models along with the hyperparameter search space. The analysis shows that a dropout rate of 0.3 provided the optimal balance between regularization and model capacity, while the learning rate of 0.001 allowed for efficient convergence. The batch size of 32 was selected based on high accuracy and training stability, and the RMSprop optimizer consistently outperformed Adam.

**Table 2:** Computational configuration of the vocal and lung model.

Parameter	Grid search range	Vocal model	Lung model
Batch size	16 and 32	32	32
Epochs	100	100	100
Dropout rate	0.3–0.4	0.3	0.3
Learning rate	0.001 and 0.01	0.001	0.001
Optimizer	Adam and RMSprop	RMSprop	RMSprop

#### Ensemble model

The predictions from the vocal and lung models are concatenated and fed into the third model, which is an ensemble learner^47^. It leverages complementary information and strengths from these two separate models, resulting in better-informed and more accurate predictions. After training the individual vocal and lung models, their respective predictions are aggregated into a single feature set to serve as the ensemble model’s input. The proposed ensemble model is a sequential model with a multi-layer architecture that takes this two-dimensional input and processes it through a dense layer with 64 units, rectified linear unit (ReLU) activation, batch normalization, and dropout layers. The final layer is a dense layer with one output unit and a sigmoid activation function. Table 3 shows the parameters used to create the ensemble model, along with its hyperparameters.

**Table 3:** Various parameters of ensemble models.

Layer name	Model hyperparameter	Output shape	Parameter
Input_layer	–	None, 2	0
Dense + normalization	Unit = 32, L1 = 0.01, L2 = 0.01	None, 32	96
Dropout	(0.4)	None, 32	0
Dense	1, activation = sigmoid, L1 = 0.01, L2 = 0.01	None, 1	33

The training of the ensemble model is enhanced through the early stopping technique. This model achieves high-accuracy classification by effectively discriminating between normal and abnormal vocal and lung data. The optimal hyperparameters for the ensemble model, including batch size, epochs, dropout rate and learning rate are determined using a grid search algorithm. Parameter selection is based on high test and validation accuracy, low test loss and optimizing training efficiency. Table 4 presents the optimal hyperparameters for the ensemble models alongside the explored hyperparameter search space. The dropout rate of 0.4 provides an optimal balance between regularization and model capacity, while the learning rate of 0.002 enables efficient convergence during the 50 epochs.

**Table 4:** Computational configuration of ensemble model.

Parameter	Grid search range	Ensemble model
Batch size	16 and 32	16
Epochs	–	50
Dropout rate	0.3–0.4	0.4
Learning rate	0.001–0.01	0.002
Optimizer	Adam and RMSprop	Adam

## Result and discussion

### Multiclass classification of vocal diseases

**Fig. 2:**
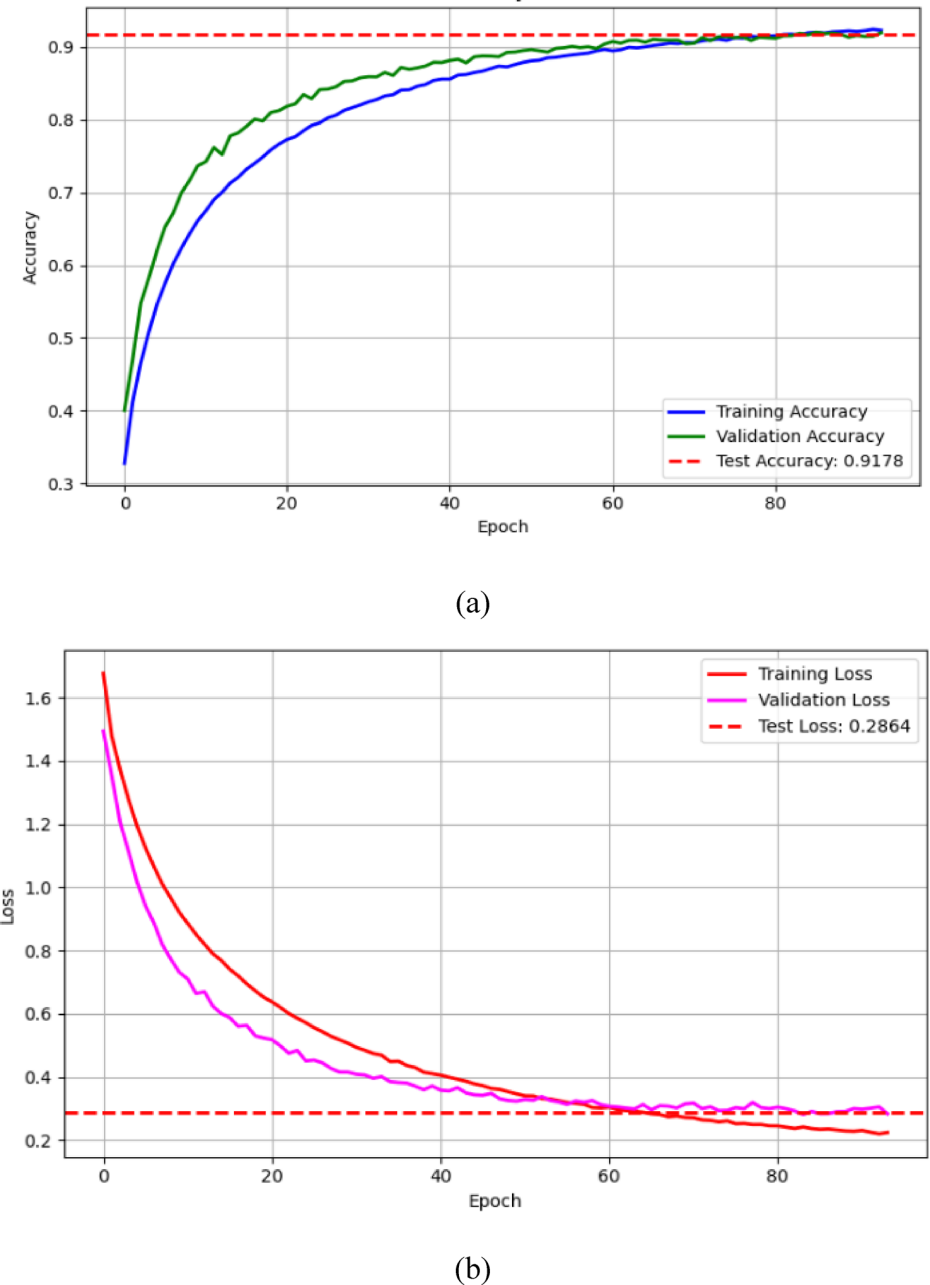
Performance evaluation of the CBiRNN model for the multiclass vocal dataset. (**a**) Training and validation accuracy curve over epochs. (**b**) Training and validation loss curve over epochs.

The proposed CBiRNN model is trained on the frequency spectrum of the vocal signal with its augmented signal to classify normal and pathological states in a multiclass setting. During the training phase, the model achieved 92% accuracy (Figure 2a) a visual representation of training accuracy over validation accuracy for the vocal model. As the number of epochs increases, accuracy also grows until it reaches the model’s maximum training limit. The close tracking between training and validation accuracy indicates no signs of overfitting. Another form of curve used to evaluate the model is the loss plot shown in Fig. 2b which depicts the statistical loss over the estimated number of epochs. The validation accuracy and loss curves are plotted alongside the training accuracy and loss curves to test for potential overfitting. If the training and validation curves have comparable patterns with no major deviations, it indicates that the proposed model is well-fitted to the input data. This demonstrates that the model can correctly classify signals into normal and five diseased situations.

**Table 5:** Performance assessment of the vocal model.

Data	Precision	Recall	F1-Score
Normal	0.87	0.79	0.83
Dysphonia	0.94	0.94	0.94
Functional dysphonia	0.88	0.95	0.91
Laryngeal nerve palsy	0.92	0.88	0.90
Laryngitis	0.95	0.96	0.96
Spasmodic dysphonia	0.95	0.99	0.97

**Fig. 3:**
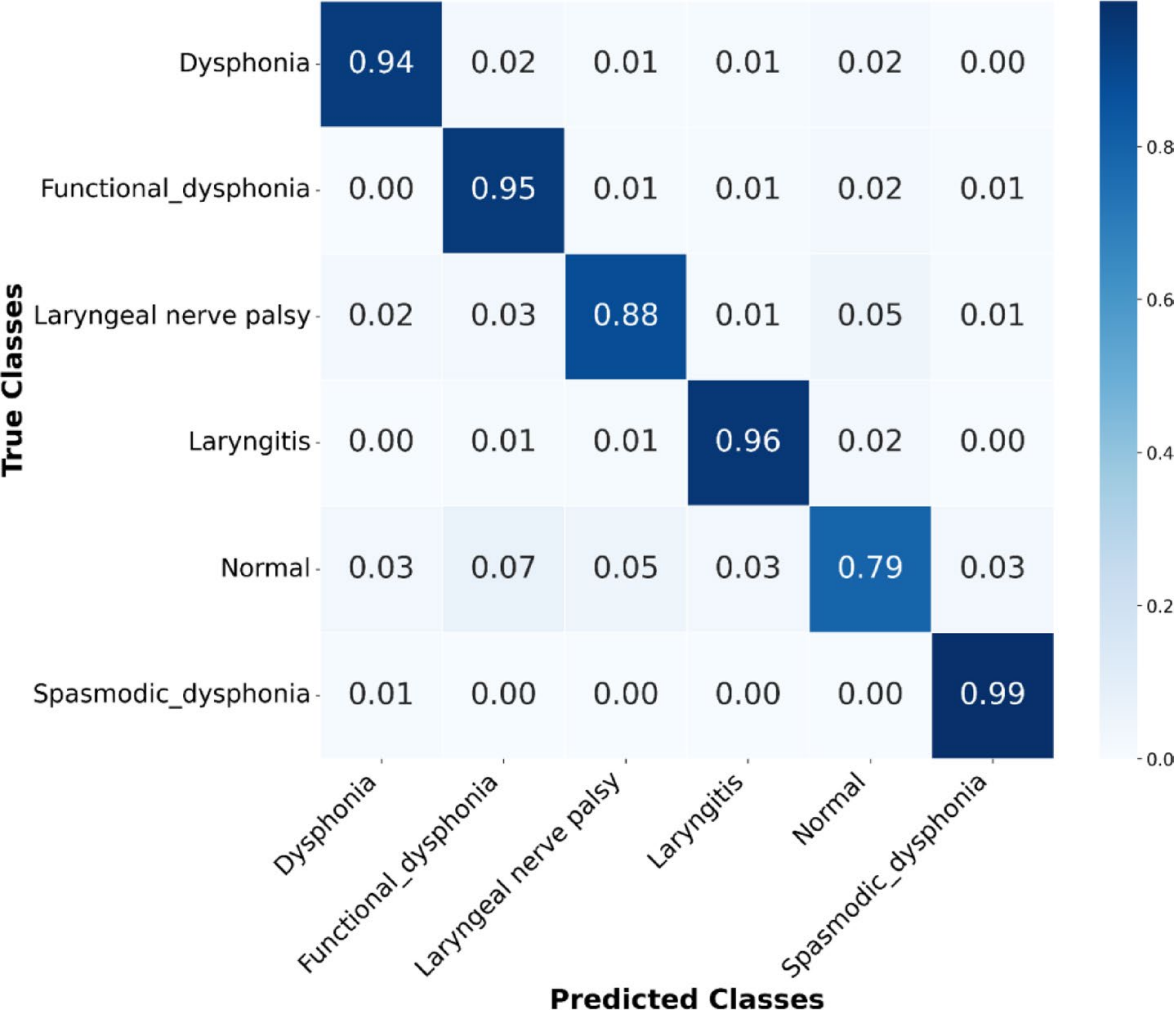
Confusion matrix for the vocal model.

The performance of the vocal model is presented in Table 5, which displays the precision, recall, and F1-score for the multiclass classification of normal and five different voice pathological conditions. The model achieves an average accuracy of 92% when classifying five different pathological cases. This model takes 2.07 s to test the entire test set of 27,021 samples, which supports our claim for real-time application viability. The model has a lightweight architecture of about 1.24 MB with 324,358 parameters and achieves high accuracy in classifying five pathological conditions with normal signals. Figure 3 depicts a normalized confusion matrix comparing testing accuracy and providing a full classification of diseases against normal and pathological situations.

### Multiclass classification of lung diseases

**Fig. 4:**
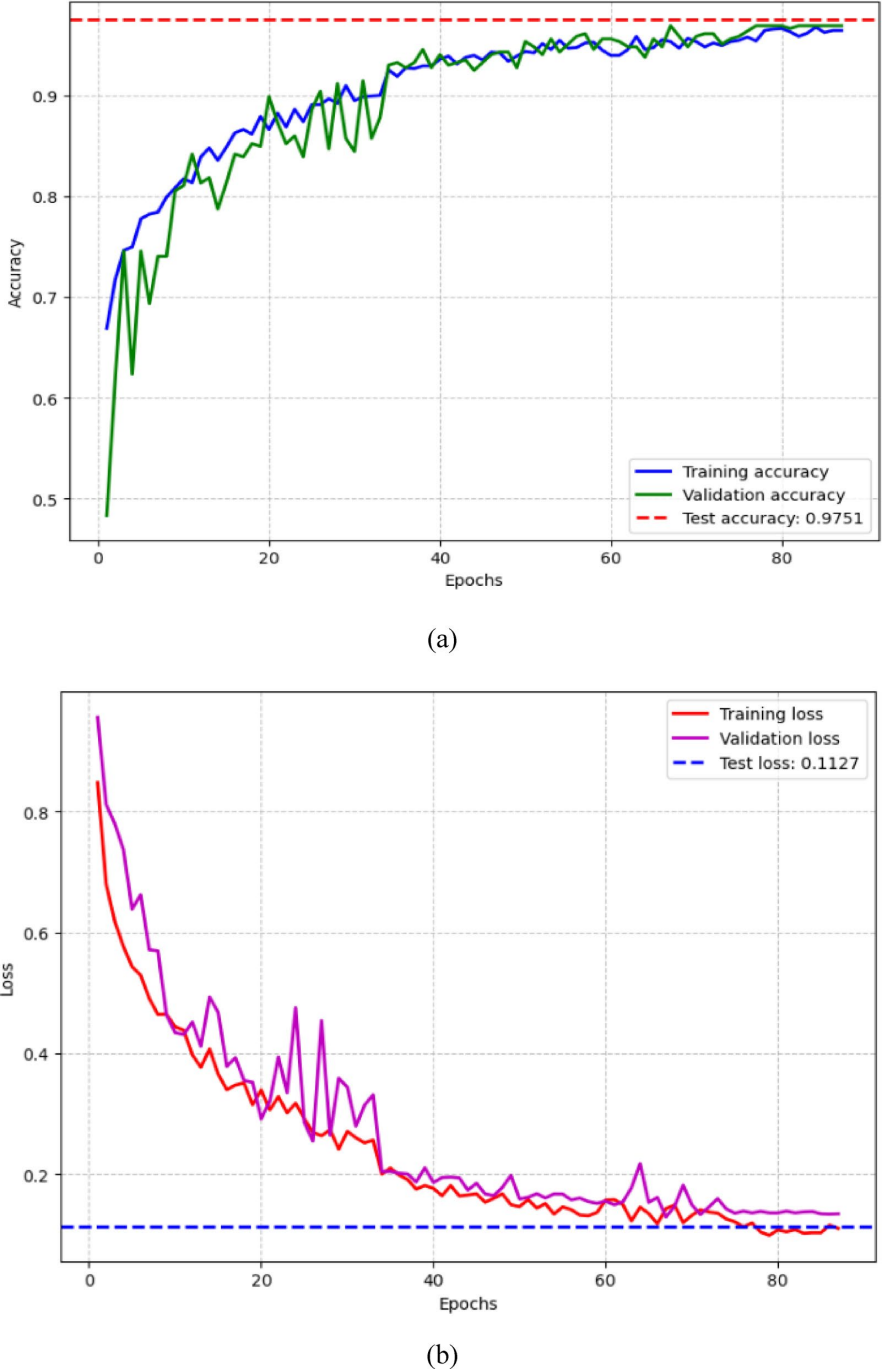
Performance evaluation of the CBiRNN model for the multiclass lung dataset. (**a**) Training and validation accuracy curve over epochs. (**b**) Training and validation loss curve over epochs.

The lung model is trained using the spectrum values of lung diseases and normal signals and its performance metrics of the model are presented in Table 6. The model achieves 98% accuracy across the three classes, which includes COPD and pneumonia as pathological cases. To validate the lung model on overfitting the Fig. 4a depicts a visual representation of training accuracy over validation accuracy for the lung model. As the number of epochs increases, accuracy also grows until it reaches the maximum limit of the model’s training and both training and validation accuracy do not have deviation. Another form of curve used to evaluate the model is the loss plot shown in Fig. 4b which depicts the statistical loss over the estimated number of epochs.

**Table 6:** Performance evaluation of lung diseases.

Data	Precision	Recall	F1-score
Normal	0.98	0.99	0.98
COPD	1.00	0.95	0.97
Pneumonia	0.95	0.99	0.97

**Fig. 5:**
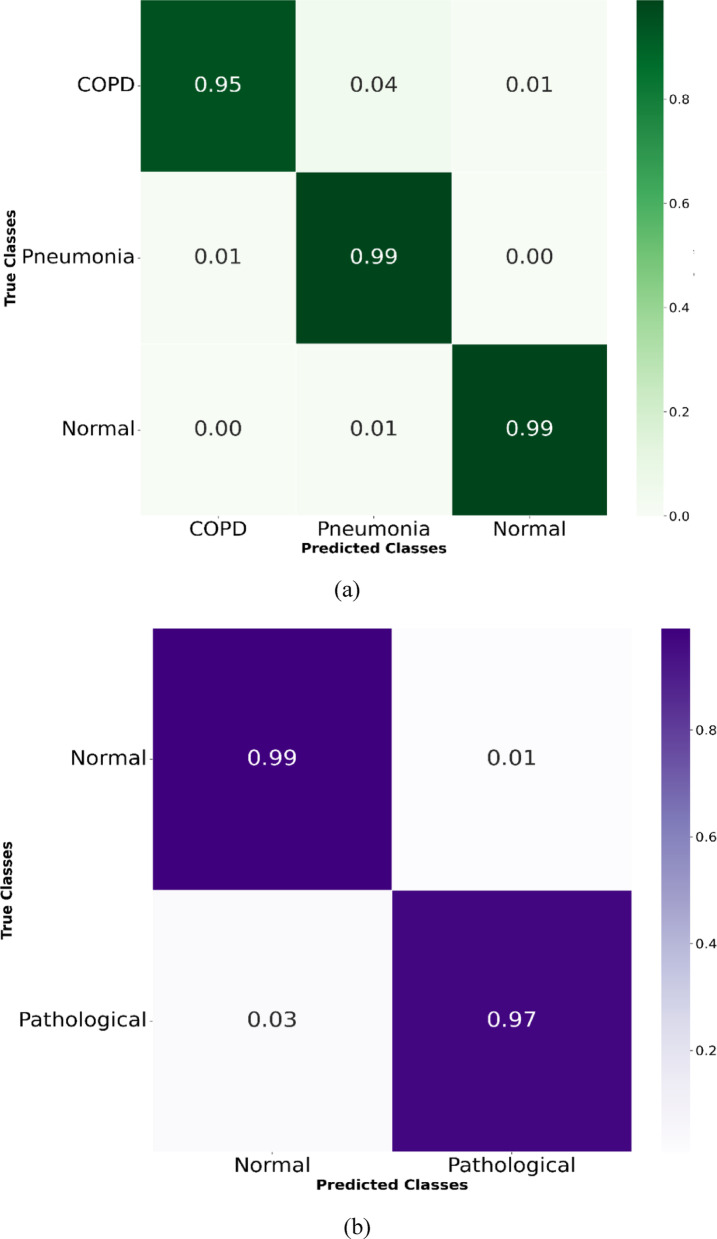
Classification performance visualization using confusion matrix. (**a**) Lung model confusion matrix showing classification across different lung conditions. (**b**) Multiple disease classification confusion matrix demonstrating overall model performance.

The model’s accuracy is evaluated using a normalized confusion matrix plot that compares normal and two diseased examples, as shown in Fig. 5a. The mean performance of both vocal and lung datasets is presented in Table 7 with 95% confidence intervals. Statistical significance testing yielded a p-value < 0.001, demonstrating highly significant performance with an accuracy of 92% in classifying 6 voice pathological conditions and an accuracy of 98% in classifying 3 lung pathological conditions.

**Table 7:** Statistical performance assessment of vocal and lung models with confidence intervals.

Metric	Vocal model		Lung model	
	Mean	Confidence intervals	Mean	Confidence intervals
Accuracy	0.918	[0.914, 0.921]	0.975	[0.960, 0.988]
Precision	0.917	[0.913, 0.921]	0.976	[0.963, 0.988]
Recall	0.918	[0.914, 0.921]	0.975	[0.960, 0.988]
F1	0.917	[0.913, 0.920]	0.975	[0.961, 0.988]

### Multiple diseases classification

**Fig. 6:**
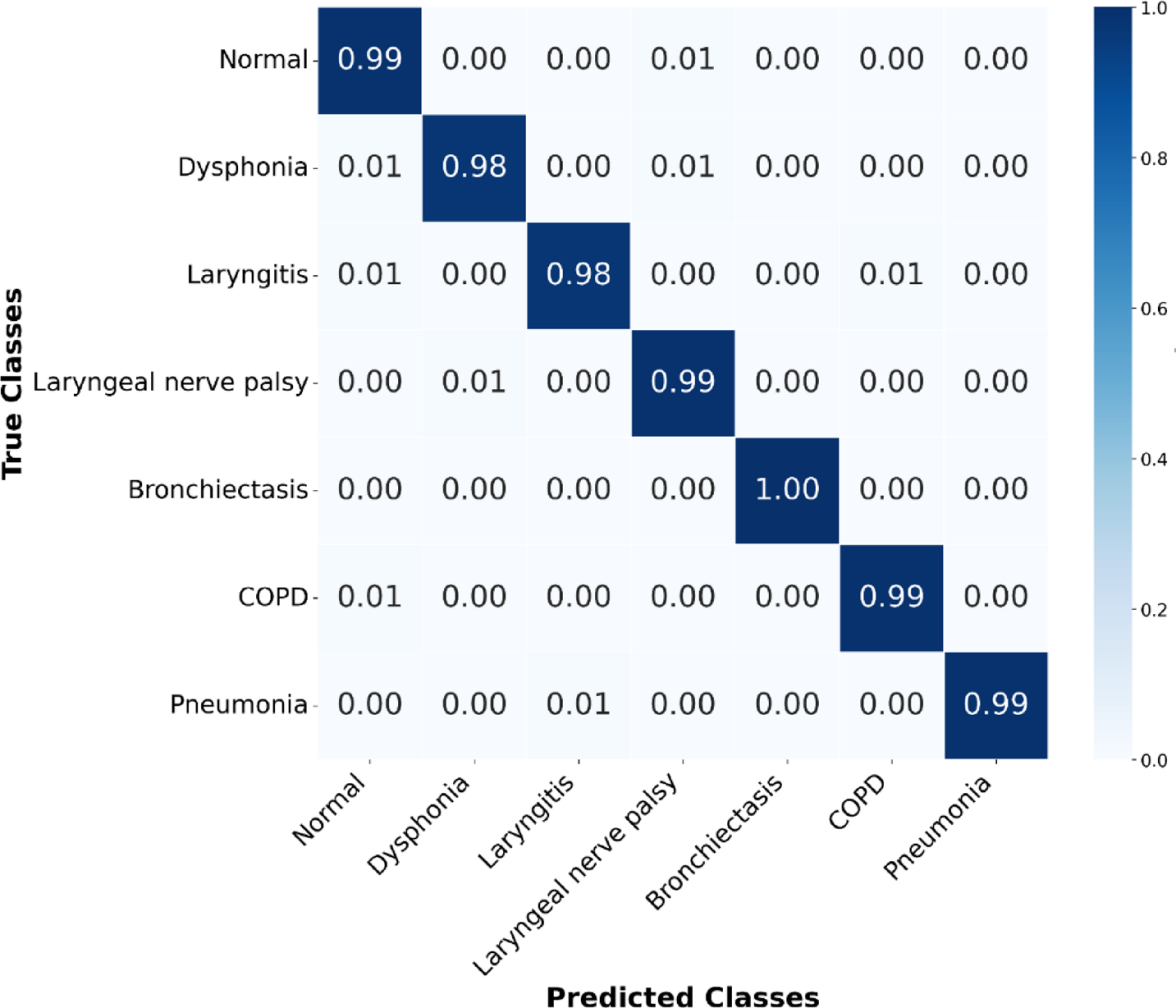
Confusion matrix for multiple pathologies and disease classification.

The prediction from the vocal and lung models is used to train the ensemble model to classify signals as normal or abnormal. The normalization confusion matrix shows that the model obtains an accuracy of more than 98% when evaluating its performance on multiple pathological cases, including lung and vocal disorders, is shown in Fig. 5b. The obtained classification accuracy outweighs the computational costs for processing sequential data. The model is then fine-tuned to distinguish between normal and pathological conditions, such as laryngeal nerve palsy, laryngitis, dysphonia, bronchiectasis, COPD, pneumonia, and abnormalities of the voice and lungs. The model achieved 98% accuracy with a well-fit model, and when fine-tuned, it shows no overfitting, resulting in very little loss and the normalized confusion matrix shown in Fig. 6 for normal and 6 pathological conditions.

## Discussion

This study highlights the enormous potential of speech signal analysis in the classification of diverse diseased signals such as vocal and lung disorders, with the suggested CBiRNN model achieving excellent accuracy in both vocal and lung pathological situations. This paper consists of three important aspects: the first model is used for multiclass classification of vocal model from the MFCC feature of speech signal with the help of deep neural network combination; the second part is focused on detection and classification of lung abnormalities by identifying the various pathological disorders; and the third part is to combine all prediction from these classes into a single abnormal and normal signal by combining all the prediction and displaying the result in form of confusion matrix.

The results obtained were compared to earlier studies that were recently proposed, and the results are displayed in Table 8. To ensure the fairness of comparison, all methods were evaluated on the SVD dataset. This comparison uses unified assessment metrics such as precision, recall, and the F1-score and these studies used several artificial intelligence algorithms to analyze data separated into six subgroups with one to five disease disorders each. Furthermore, these studies were conducted using the CNN^30^, CNN and LSTM^25^, spectrogram transformer^31^, and OpenL3 SVM^27^ model to extract features such as entropy, zero crossing rate (ZCR)^28^, Mel frequency cepstral coefficient^29^, skewness, kurtosis, shimmer, jitter, root mean square error (RMSE), segmentation, and Mel spectrogram.

In this study, our CBiRNN model demonstrates superior performance across the board for SVD dataset achieving a high F1-score of 92% in multi-class voice pathology classification. Table 9 presents additional investigations conducted using the Respiratory Sound Database for lung pathology diagnosis. This study employed various deep learning architectures including CNN^37,38^, LSTM^34^, and ConvLSTM^36^ with speech signal features extracted using MFCC, ZCR, chroma^39^, centroid, skewness, kurtosis, Tonnetz, contrast, and Wavelet transform^40^. Model performance was evaluated using precision, recall, and F1-score metrics. The proposed CBiRNN model achieves 98% accuracy across all three classes (Normal, COPD, and Pneumonia) and requires only 0.83 s for model testing. It demonstrates a good balance between sensitivity and specificity for both normal and pathological cases, outperforming other architectures explored during the research.

**Table 8:** Comparative performance analysis of vocal pathology.

Ref	Feature	Model	Classes	Precision	Recall	F1-score
SVD, VD, 2021^28^	entropy, zero crossing rates (ZCR), Mel frequency cepstral coefficients (MFCC), Skewness, Kurtois, Shimmer and Jitter	CNN and LSTM	Normal	0.7	0.7	0.69
			Dysphonia	0.79	0.81	0.8
			Functional dysphonia	0.75	0.77	0.78
			Laryngeal nerve palsy	0.81	0.82	0.81
			Laryngitis	0.82	0.85	0.84
			Spasmodic dysphonia	0.87	0.87	0.9
SVD, 2022^25^	ZCR, RMSE, MFCCs	CNN-RNN	Normal	0.79	0.83	0.82
			Dysphonia	0.87	0.89	0.88
			Functional dysphonia	0.85	0.87	0.87
			Laryngeal nerve palsy	0.89	0.84	0.85
			Laryngitis	0.85	0.89	0.89
			Spasmodic dysphonia	0.91	0.9	0.92
SVD, 2023^29^	MFCC and LPC	SMOTE and CNN	Normal	0.74	0.75	0.72
			Dysphonia	0.85	0.8	0.83
			Functional dysphonia	0.88	0.85	0.86
			Laryngeal nerve palsy	0.72	0.76	0.79
			Laryngitis	0.79	0.8	0.8
			Spasmodic dysphonia	0.8	0.84	0.82
SVD, 2023^30^	-	CNN	Normal	0.62	0.59	0.69
			Dysphonia	0.82	0.84	0.8
			Functional dysphonia	0.85	0.89	0.78
			Laryngeal nerve palsy	0.71	0.7	0.72
			Laryngitis	0.8	0.81	0.81
			Spasmodic dysphonia	0.85	0.85	0.88
VOICED, 2023 ^27^	Mel spectrogram	OpenL3-SVM	Normal	0.78	0.83	0.82
			Dysphonia	0.85	0.9	0.89
			Functional dysphonia	0.85	0.87	0.87
			Laryngeal nerve palsy	0.89	0.83	0.85
			Laryngitis	0.9	0.89	0.9
			Spasmodic dysphonia	0.89	0.9	0.92
Real time data, 2024^31^	Segmentation	Audio spectrogram transformer	Normal	0.77	0.78	0.74
			Dysphonia	0.83	0.88	0.87
			Functional dysphonia	0.86	0.87	0.89
			Laryngeal nerve palsy	0.83	0.81	0.82
			Laryngitis	0.85	0.87	0.89
			Spasmodic dysphonia	0.9	0.89	0.91
Proposed work	MFCC	CBiRNN	Normal	0.87	0.79	0.83
			Dysphonia	0.94	0.94	0.94
			Functional dysphonia	0.88	0.95	0.91
			Laryngeal nerve palsy	0.92	0.88	0.90
			Laryngitis	0.95	0.96	0.96
			Spasmodic dysphonia	0.95	0.99	0.97

**Table 9:** Comparative performance analysis of lung pathology.

Ref	Feature	Model	Classes	Precision	Recall	F1-score
ICBHI Respiratory database, 2020^32^	EMD, DWT	quadratic discriminant	Normal	0.93	0.93	0.94
			COPD	0.94	0.95	0.96
			Pneumonia	0.89	0.93	0.91
Real time, 2021^34^	MFCC	LSTM	Normal	0.92	0.96	0.97
			COPD	0.94	0.95	0.93
			Pneumonia	0.89	0.88	0.89
ICBHI, Coswara, 2022^35^	MFCC	CRNN	Normal	0.96	0.97	0.97
			COPD	0.96	0.97	0.96
			Pneumonia	0.87	0.88	0.89
ICBHI Respiratory database, Coswara, 2022^38^	ZCR, chroma, centroid, spread, skewness, kurtosis, MFCC	CNN	Normal	0.91	0.89	0.92
			COPD	0.83	0.85	0.86
			Pneumonia	0.9	0.94	0.91
ICBHI, 2022^39^	MFCC, Mel-spec, chroma	CNN	Normal	0.95	0.96	0.94
			COPD	0.93	0.95	0.96
			Pneumonia	0.9	0.89	0.9
Respiratory Database @TR, 2023^37^	MFCC, Chroma, Mel-spec, Tonnetz, contrast	LSTM	Normal	0.94	0.95	0.9
			COPD	0.9	0.88	0.93
			Pneumonia	0.85	0.9	0.91
ICBHI, 2024^40^	Mel spec, CWT	Conv autoencoder	Normal	0.92	0.94	0.91
			COPD	0.93	0.95	0.97
			Pneumonia	0.89	0.88	0.89
Real time, 2024^36^	CNN and LSTM	ConvLSTM	Normal	0.98	0.96	0.97
			COPD	0.9	0.91	0.92
			Pneumonia	0.92	0.95	0.94
Proposed work	MFCC	CBiRNN	Normal	0.98	0.99	0.98
			COPD	1.00	0.95	0.97
			Pneumonia	0.95	0.99	0.97

**Fig. 7:**
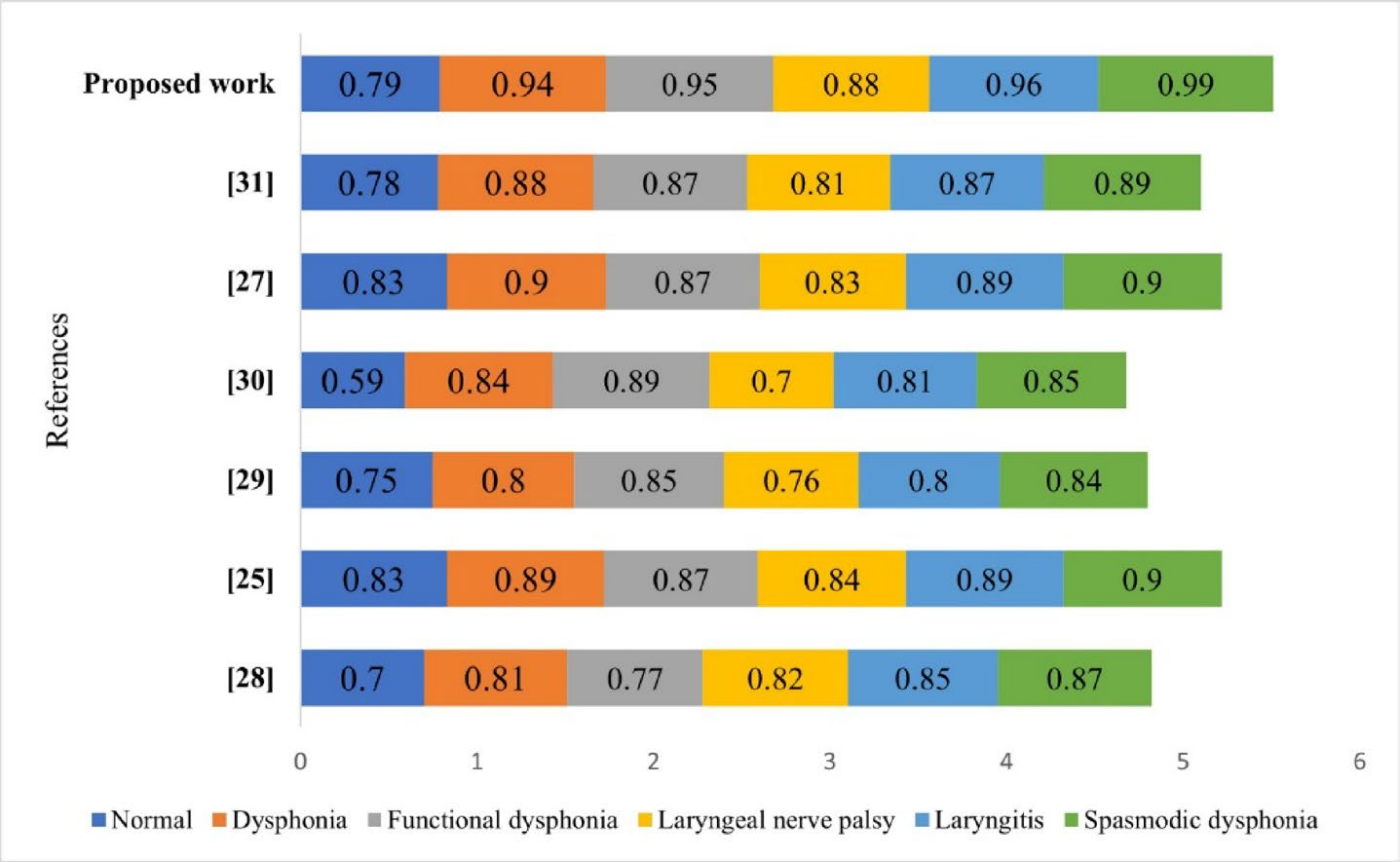
Comparison of performance metrics across various voice disorder classifications.

The horizontal stacked bar chart in Fig. 7 used to display vocal diseases classification for six different studies, with the Proposed work. The proposed work shows superior results compared to previous research labeled as references^25,27–31^. Each colored segment represents a different voice condition: normal speech (blue), dysphonia (orange), functional dysphonia (grey), laryngeal nerve palsy (yellow), laryngitis (light blue), and spasmodic dysphonia (green). The performance values range from approximately 0.59 to 0.99, with the proposed work achieving the highest scores in most categories, particularly excelling in spasmodic dysphonia detection (0.99) and laryngitis (0.96).

**Fig. 8:**
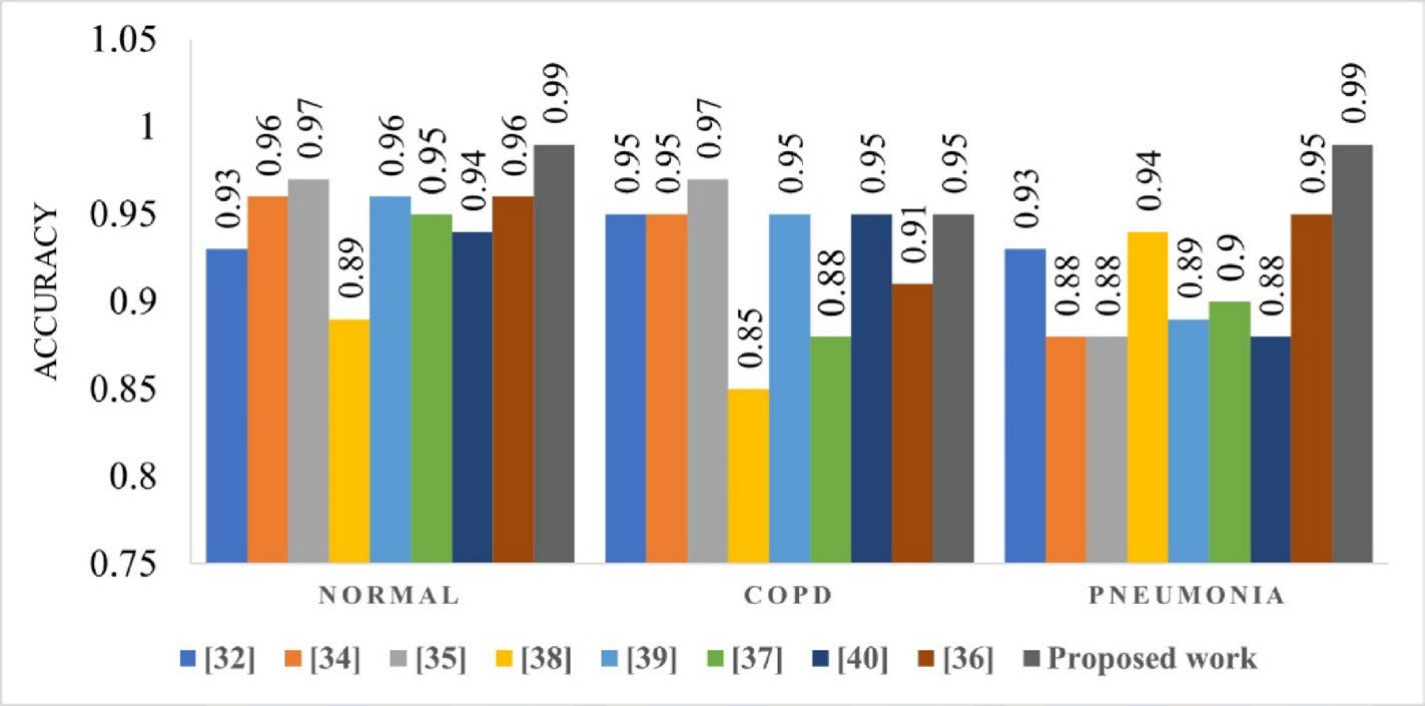
Comparison of performance metrics across various lung disorder classifications.

Figure 8 represents the clustered column chart, which displays a comparison of various lung models for classifying respiratory conditions across three categories: Normal, COPD (Chronic Obstructive Pulmonary Disease), and Pneumonia.

 The grouped bar chart presents performance metrics ranging from 0.75 to 1.05 on the vertical axis. Each colored bar represents a different referenced work^32,34–40^ or the Proposed work, shown in dark gray. The Proposed work consistently achieves among the highest scores across all three conditions, matching or outperforming previous methods with values of 0.99 for Normal and Pneumonia and 0.95 for COPD. Performance appears generally strongest for Normal classification across most methods, while Pneumonia classification shows more variability among the different approaches.

## Conclusion and future work

This research presents a hybrid approach for health monitoring by integrating vocal and lung signal analysis using a hybrid CBiRNN model. To examine different data sources, signal augmentation techniques and MFCCs are used for feature extraction. The ensemble approach improves prediction accuracy by combining complementary information from vocal and lung signals, achieving 98% of accuracy. Using this trained model, clinicians can predict patient voice and lung diseases with the help of speech data obtained from a microphone. The model prediction is given in the form of a confusion matrix, which consists of normal and pathological labels, and the diagonal in the confusion matrix indicates the medical condition of the patient. This prediction enables clinicians to diagnose diseases easily in a non-invasive way. This integrated analysis not only increases abnormality identification but also provides a more complete picture of physiological conditions. The suggested multimodel processing technique has the potential to accelerate the development of advanced health tracking systems, paving the way for more dependable and accurate diagnostics. This multi-network model currently focuses only on classifying two pathological conditions. In future work, the model will be developed further to classify different pathological conditions, and efforts will be made to improve its accuracy.

## Data Availability

Data is available in github linkhttps://github.com/Revathisankarv/Deep-learning-model-for-multimodal-pathological-analysis/tree/main.
